# Myeloid-derived suppressor cells and regulatory T cells in colorectal cancer: a synergistic immunosuppressive axis and emerging therapeutic opportunities

**DOI:** 10.3389/fimmu.2026.1757513

**Published:** 2026-01-29

**Authors:** Wenxing Zhang, Chenrui Jin, Shuyuan Liu, Xing Wan, Yu Li, Jifeng Liu, Zhijun Duan, Jingyuan Ma, Yunhai Gao

**Affiliations:** 1 The First Clinical Medical College, Liaoning University of Traditional Chinese Medicine, Shenyang, China; 2 Department of Proctology, The First Affiliated Hospital of Liaoning University of Traditional Chinese Medicine, Shenyang, China; 3 Department of General Surgery, The First Affiliated Hospital of Liaoning University of Traditional Chinese Medicine, Shenyang, China; 4 Institute of Integrative Medicine, Dalian Medical University, Dalian, China; 5 Department of General Surgery, The First Affiliated Hospital of Dalian Medical University, Dalian, China; 6 Department of Gastroenterology, The First Affiliated Hospital of Dalian Medical University, Dalian, China

**Keywords:** cold tumor, colorectal cancer, gut microbiota, immune checkpoint inhibitors, MDSC, microsatellite stability, regulatory T cell

## Abstract

Microsatellite-stable (MSS)/proficient mismatch-repair (pMMR) colorectal cancer (CRC) accounts for more than 85% of cases but responds poorly to single-agent immune checkpoint inhibitors (ICIs), with objective response rates remaining below 5%. A principal barrier to effective immunotherapy in these tumors is a durable immunosuppressive axis formed by myeloid-derived suppressor cells (MDSCs) and regulatory T cells (Tregs) within the tumor microenvironment. This axis impedes antitumor immunity through multilayered mechanisms including bidirectional chemotactic recruitment, reciprocal cytokine signaling, metabolic suppression and exosome-mediated communication. CRC is uniquely influenced by the gut microbiota: Fusobacterium nucleatum promotes MDSC/Treg enrichment via TLR4–NF-κB and Fap2–TIGIT pathways; Peptostreptococcus anaerobius acts through integrin–PI3K–NF-κB signaling; and microbial metabolites such as 4-HPA activate JAK2/STAT3–CXCL3 signaling to expand MDSC populations. Concurrently, a hypoxia–lactate–HIF-1α–CD73/A2AR circuit further stabilizes suppressive phenotypes, forming a “microbiota–metabolism–hypoxia–MDSC–Treg” cascade. Emerging clinical and translational data indicate that disrupting this axis can sensitize MSS-CRC to ICIs: for example, Zanzalintinib combined with Atezolizumab reported survival benefit in the STELLAR-303 trial, and dual blockade of novel checkpoints with PD-(L)1 has been associated with enhanced immune activation in solid tumors. Targeting the MDSC–Treg axis therefore represents a promising strategy to overcome immunotherapy resistance in MSS/pMMR CRC.

## 1 Introduction

Colorectal cancer (CRC) remains one of the most prevalent malignancies worldwide, with roughly 1.9 million new cases diagnosed in 2022, ranking third in incidence and second in cancer-related mortality (1). Surgery and chemoradiotherapy continue to form the backbone of treatment, yet the introduction of immune checkpoint inhibitors (ICIs) has reshaped the therapeutic landscape and marked a major step toward precision immunotherapy in CRC (2). Despite recent progress, responses to ICIs are still very different among CRC subtypes. Patients with mismatch-repair–deficient (dMMR) or microsatellite instability–high (MSI-H) tumors often benefit from ICIs, but patients with microsatellite-stable (MSS) or mismatch-repair–proficient (pMMR) disease, who make up about 85% of all CRC cases, show response rates below 5% (3, 4). This sharp difference shows that the tumor microenvironment (TME) plays a key role. Inside the TME, strong immunosuppressive signals act as major barriers to treatment (5).

Within this suppressive landscape, myeloid-derived suppressor cells (MDSCs) and regulatory T cells (Tregs) stand out as dominant contributors to CRC immune tolerance (6). MDSCs impair effector T-cell activity through mechanisms involving arginase-1 (ARG1), inducible nitric oxide synthase (iNOS), reactive oxygen species (ROS), and PD-L1 (7). Tregs further weaken antitumor immunity through CD25, CTLA-4, IL-10, and TGF-β (8, 9). Recent studies show that these two cell types rarely act alone. Instead, they form a close “MDSC–Treg axis” that depends on mutual recruitment, cytokine signals, metabolic support, and epigenetic changes (6). This axis helps build a TME that responds poorly to ICIs.

The biological features of CRC make this suppressive axis even stronger. The constant interaction between the gut microbiota and the intestinal immune system produces many regulatory signals. For example, Fusobacterium nucleatum increases MDSC and Treg buildup through TLR4–NF-κB signaling and the Fap2–TIGIT pathway (10, 11). Long-lasting inflammation and continuous IL-6/STAT3 activation push myeloid cells toward suppressive types (12). In addition, tumor-related metabolites such as lactate, adenosine, and kynurenine build up in the TME and support both the energy needs and the signaling pathways of MDSCs and Tregs (13). Taken together, understanding how the MDSC–Treg axis starts and works is important for explaining immune resistance in pMMR/MSS CRC.

This review brings together current knowledge on how these cells are recruited, how they communicate, how their metabolism is regulated, and how feedback loops maintain the axis. It also summarizes therapeutic strategies that aim to break this suppressive network and highlights new progress in microbiota-based treatments, metabolic targeting, extracellular-vesicle studies, and spatial multi-omics. By connecting evidence from cellular, metabolic, and microbial research, we hope to offer a clear view of the immunosuppressive structure of CRC and to support future precision-immunotherapy approaches for MSS/pMMR disease.

## 2 Enrichment, recruitment, and mutual induction of MDSCs and Tregs in CRC

MDSCs come from disrupted myeloid development and form a broad suppressive population. MDSCs comprise two primary subsets: polymorphonuclear MDSCs (PMN-MDSCs) and monocytic MDSCs (M-MDSCs) (14). In the CRC tumor microenvironment, M-MDSCs exhibit substantial phenotypic and functional overlap with M2-polarized tumor-associated macrophages (TAMs), characterized by high expression of CD206, ARG1, and IL-10. Tumor-derived factors, including colony-stimulating factor-1 (CSF-1), IL-6, and TGF-β, frequently drive M-MDSCs to differentiate into immunosuppressive M2-like TAMs, amplifying angiogenesis, tissue remodeling, and immune evasion (15). Treg–MDSC engagement further reinforces this suppressive myeloid lineage: Tregs secrete IL-10 and TGF-β, which upregulate ARG1 and iNOS in MDSCs while inhibiting their maturation into immunostimulatory dendritic cells (DCs) (15). This blockade of DC differentiation—mediated in part by reduced MHC II expression and impaired antigen-presenting capacity—prevents effective T-cell priming and sustains the tolerogenic TME in CRC. Their levels are high in both the blood and the tumor tissues of CRC patients, and their numbers increase with disease stage and metastasis (16). Tregs, marked by FoxP3, help keep immune balance and prevent autoimmunity (17). In CRC, MDSCs and Tregs often increase together. This pattern is not only a spatial feature but also a sign that these cells form a coordinated suppressive partnership. Chemokines, cytokines, and direct cell-to-cell signals work together to promote their recruitment and mutual induction. This section explains how these cells gather inside the tumor, become activated, and form a functional connection.

### 2.1 Chemokine-driven bidirectional recruitment

The chemokine environment in CRC guides both MDSCs and Tregs toward the tumor (18). Tumor cells, stromal cells, and immune cells release CCL2, CCL3, CCL4, CCL5, CXCL8, and other chemokines that create gradients for cell movement (19). MDSCs strengthen this process. They produce CCL3, CCL4, and CCL5, which attract CCR5^+^ Tregs and increase their buildup in the tumor (20). MDSC-derived S100A8/A9 also helps keep MDSCs coming in and affects how other immune cells move (21). Tregs add to this loop. They do not directly recruit MDSCs, but they secrete CCL22 to support CCR4^+^ Treg buildup and release IL-10 and TGF-β, which increase CXCL8-driven recruitment of CXCR2^+^ PMN-MDSCs (22, 23).

These chemokine signals work together to form a positive-feedback loop. CCR5 ligands from MDSCs and chemokines from Tregs maintain constant recruitment (24). *In vivo*, higher levels of CCL4/CCL5 lead to more Treg infiltration, and CCR5 loss reduces Treg buildup and weakens immunosuppression (24). In short, chemokine-mediated recruitment is the first step in forming the MDSC–Treg axis and sets up close spatial contact between the two cell types (20).

### 2.2 MDSC-induced Treg expansion and Treg-supported MDSC function

Once they are located in the same area, MDSCs and Tregs strengthen each other through soluble factors and direct contact. This cooperation helps maintain long-term immune tolerance in CRC (25). MDSCs strongly promote Treg expansion. High levels of ARG1 and iNOS change the metabolic environment, limit effector T-cell growth, and push CD4^+^ T cells to become FoxP3^+^ Tregs (26, 27). MDSCs also produce TGF-β and IL-10, which are essential for peripheral Treg induction (14). Engagement of PD-1 by PD-L1 on MDSCs further dampens T-cell activation and promotes tolerance (28). Although M-MDSCs retain partial antigen-presenting capacity, insufficient co-stimulation results in low-reactivity or regulatory T-cell outcomes (29). Consistently, tissue studies demonstrate a strong correlation between MDSC infiltration and intratumoral FoxP3^+^ Treg abundance (30, 31).

Tregs, in turn, sustain and enhance MDSC suppressive activity. Their secretion of IL-10, TGF-β, and IL-35 activates key pathways in MDSCs and increases ARG1 and iNOS expression, strengthening their ability to suppress effector T cells (32, 33). Through CTLA-4-mediated interactions with dendritic cells, Tregs reduce co-stimulatory signaling and induce IDO, indirectly creating a tolerogenic microenvironment favorable to both cell types (34). In addition to these cytokine- and DC-dependent mechanisms, CD40–CD40L engagement provides a critical membrane-bound licensing signal: MDSCs in CRC exhibit high CD40 expression, and interaction with CD40L on activated Tregs drives NF-κB–STAT3 activation and upregulates IL-10, TGF-β, ARG1, and iNOS, thereby amplifying both Treg induction and MDSC suppressive function. Blockade of CD40 or CD40L has been shown to disrupt this reciprocal reinforcement and restore antitumor T-cell activity in multiple tumor models (25, 35).

Beyond cytokine signaling and metabolic suppression, direct contact further stabilizes this crosstalk. β_2_ integrins mediate physical interactions between MDSCs and Tregs, enhancing their suppressive output and facilitating immune evasion (6). Transcriptomic analyses show that this suppressive circuit appears early in CRC development and can already be found in nearby mucosal tissues. This means the MDSC–Treg axis begins to change local immune balance at the very early stages of tumor formation (36, 37). These mechanisms work together to form a strong positive-feedback system that keeps chronic immunosuppression in the CRC microenvironment.

### 2.3 Treg subsets, phenotypic plasticity, and the role of Th17 cells in CRC immunosuppression

Tregs in the CRC TME display marked heterogeneity and phenotypic plasticity, which amplify their contribution to immunosuppression beyond a uniform suppressive role (38). Human FOXP3^+^ Tregs subdivide into naïve-like central Tregs, with the latter showing stronger suppressive activity and predominant enrichment in CRC tissues (39). Furthermore, tumor-infiltrating Tregs frequently co-express helper lineage-specific transcription factors, generating “Th-like” hybrids such as RORγt^+^ Th17-like Tregs or T-bet^+^ Th1-like Tregs (40). This adaptability enables Tregs to respond to inflammatory signals in the TME, stabilizing their suppressive phenotype while retaining high FOXP3 levels.

A prominent manifestation of Treg plasticity in CRC is the generation of ex-Th17 Tregs, derived from Th17 precursors that acquire a FOXP3^+^ suppressive profile (41, 42). These cells, including IL-17A^+^FOXP3^+^ intermediates or IL-17A^−^FOXP3^+^ derivatives, preserve some proinflammatory traits but primarily mediate immunosuppression, facilitating tumor progression (43). Such transdifferentiation occurs in TGF-β-rich microenvironments, where Th17 cells—originally polarized by IL-6 and IL-23—downregulate RORγt and induce FOXP3 expression (44).

Th17 cells exhibit a dual, often protumorigenic influence in CRC pathogenesis and frequently intersect with the MDSC–Treg axis (45). Within the CRC tumor microenvironment, Th17-derived IL-17A promotes the recruitment and activation of immunosuppressive populations, particularly PMN-MDSCs via CXCL1/CXCL2 or IL-8–CXCR2 signaling, thereby reinforcing immune evasion (46). During CRC progression, Th17/Treg balance is progressively skewed toward Treg dominance through plasticity-driven reprogramming, establishing a self-sustaining suppressive circuit (47). Notably, γδT17 cells—one of the major sources of IL-17A in human CRC—further potentiate this axis by driving PMN-MDSC mobilization and expansion (46).

**Figure 1:**
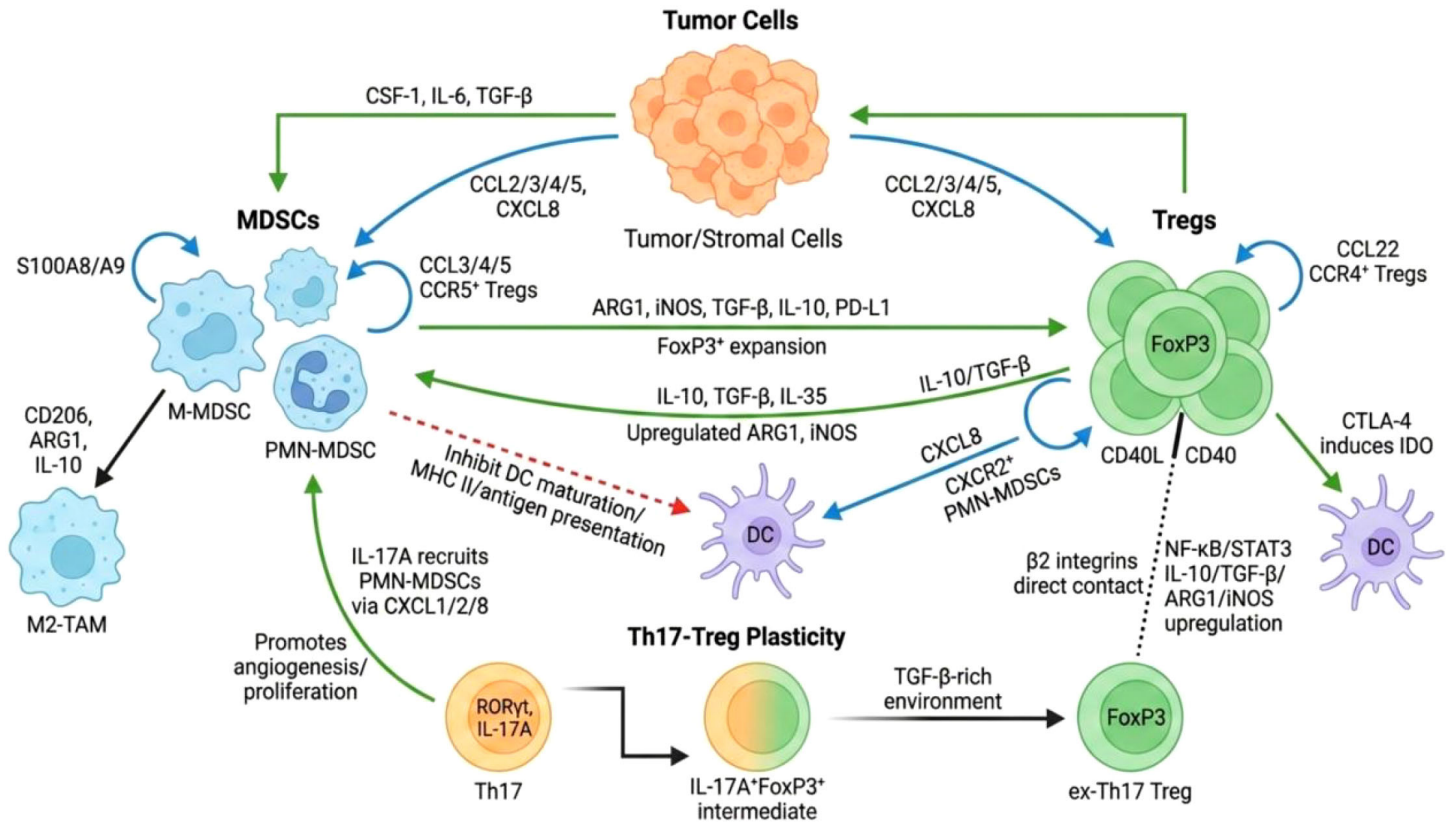
Chemokine-driven recruitment, mutual induction, and Th17-integrated plasticity of the MDSC–Treg immunosuppressive axis in CRC. Created with BioRender.com.

Collectively, these dynamics highlight that the MDSC–Treg axis integrates Th17-associated plasticity and inflammatory pathways, adding multifaceted mechanisms of immune escape in CRC, as schematically illustrated in **Figure 1**.

Tumor- and stromal-derived factors (CSF-1, IL-6, TGF-β) drive M-MDSC differentiation into M2-like TAMs (CD206^+^, ARG1^+^, IL-10^+^). Bidirectional chemokine loops (CCL2/3/4/5 from tumor/MDSCs to CCR5^+^ Tregs; CCL22, IL-10/TGF-β from Tregs enhancing CXCL8 for CXCR2^+^ PMN-MDSCs; S100A8/A9 self-recruitment) establish spatial colocalization. MDSCs promote FoxP3^+^ Treg expansion via ARG1/iNOS metabolic depletion, TGF-β/IL-10, and PD-L1/PD-1 signaling, while inhibiting DC maturation (↓MHC II). Tregs enhance MDSC suppression through IL-10/TGF-β/IL-35 (↑ARG1/iNOS), CTLA-4–DC–IDO axis, CD40–CD40L–NF-κB/STAT3, and β_2_ integrin contacts. Th17 plasticity generates ex-Th17 Tregs (TGF-β-driven RORγt↓/FoxP3↑ from IL-6/IL-23-polarized precursors), with IL-17A recruiting PMN-MDSCs (CXCL1/2/8) and promoting angiogenesis. MDSCs provide rapid, non-specific suppression (ARG1/iNOS/ROS), complemented by Treg antigen-specific, plastic tolerance, forming a multilayered network in CRC.

## 3 Molecular mechanisms by which the MDSC–Treg axis drives immunosuppression

After spatial co-localization and mutual induction, MDSCs and Tregs consolidate their suppressive partnership through cytokine signaling, metabolic regulation, contact-dependent pathways, and intercellular communication. This section explains how these mechanisms come together to build a long-lasting immunosuppressive program in CRC and allow ongoing immune escape.

### 3.1 Cytokines and coinhibitory signals as core drivers of the suppressive circuit

Cytokines and coinhibitory receptors form the main signaling loop that links MDSCs and Tregs. IL-10, TGF-β, PD-L1, and CTLA-4 are the key factors that keep immune tolerance in place (48).

As described in detail in Section 2.2, IL-10 and TGF-β serve as central bidirectional mediators of the MDSC–Treg axis. IL-10 enhances MDSC suppressive function via the JAK–STAT3 pathway, upregulating ARG1 and iNOS while impairing dendritic cell antigen presentation (26, 27, 49), and simultaneously promotes peripheral Treg induction (50, 51).

TGF-β keeps FoxP3 expression active through SMAD2/3 and reduces the cytotoxic activity of CD8^+^ T cells and NK cells (52, 53). Blocking TGF-β can improve antitumor immunity, but systemic inhibition may cause strong inflammatory side effects (54).

Contact-dependent signals also strengthen the suppressive axis. PD-L1 on MDSCs limits effector T-cell activation through PD-1 and helps Treg expansion grow (55). CTLA-4 on Tregs competes with CD80/CD86, lowers co-stimulation, induces IDO in antigen-presenting cells, and reduces effector T-cell responses (56, 57). β_2_ integrins on both MDSCs and Tregs help them stay in close contact and remain inside the tumor, which forms a “closed” immunosuppressive space (58).

### 3.2 Metabolic reprogramming as a sustaining force for immunosuppression

The CRC microenvironment shows major metabolic changes that MDSCs and Tregs use to keep long-term suppression. Arginine and tryptophan metabolism, adenosine production, and lactate buildup are central parts of this process.

High ARG1 in MDSCs reduces extracellular arginine. This weakens TCR signaling and causes effector T-cell dysfunction (59). Tryptophan breakdown through the IDO–kynurenine pathway activates AhR, increases FoxP3 transcription, and raises peripheral Treg numbers (60–62). CTLA-4 on Tregs also increases IDO expression in antigen-presenting cells, which keeps this metabolic loop active (63).

In many tumors, MDSCs and Tregs use CD39 and CD73 to make immunosuppressive adenosine from extracellular ATP (64). CD39 turns ATP/ADP into AMP, and CD73 converts AMP into adenosine (65). The accumulated adenosine then activates A2A receptors on effector T cells and NK cells. This triggers cAMP–PKA signaling and reduces activation, proliferation, and cytotoxicity in these cells (66, 67). At the same time, adenosine supports the survival and stability of suppressive immune cells, including MDSCs and Tregs, and shapes the TME into a suppressive niche (65, 68). Hypoxia in the TME increases CD39/CD73 expression through HIF-1α and raises adenosine levels even more, which strengthens immunosuppression (69). Furthermore, HIF-1α signaling can induce expression of ENTPD2 (also known as CD39L1), an ectoenzyme that converts extracellular ATP to 5’-AMP, thereby preserving MDSC suppressive phenotypes by impairing their differentiation into mature immunostimulatory dendritic cells—a mechanism that contributes to sustained myeloid immunosuppression in hypoxic solid tumors (70).

### 3.3 Key signaling pathways and epigenetic programs

Inflammation, metabolic stress, and tumor-derived signals come together at a set of transcriptional and epigenetic regulators. STAT3, NF-κB, and HIF-1α are the main factors that integrate these signals and help maintain stable suppressive phenotypes in MDSCs and Tregs.

STAT3, activated by IL-6, IL-10, GM-CSF and other upstream signals, is essential for the suppressive differentiation of MDSCs. It induces ARG1, iNOS, and PD-L1, promotes metabolic rewiring, and biases myeloid cells toward suppressive lineages (71, 72). STAT3 also modulates DNA methylation and histone acetylation to stabilize suppressive transcriptional programs in both MDSCs and Tregs (73–75). Pharmacologic STAT3 inhibition reduces the accumulation of both populations and restores CD8^+^ T-cell activity (76, 77). Moreover, the CCL2–STAT3 axis directly enhances PMN-MDSC suppressive function (78), further highlighting STAT3 as a central driver of the MDSC–Treg circuit (79).

NF-κB activation downstream of TLR4 and TNFR2 induces IL-1β, IL-6, CCL2, and CXCL8, promoting continuous recruitment and activation of MDSCs and Tregs (80, 81). Together with STAT3, NF-κB increases S100A8/A9 and PD-L1 expression, intensifying immune evasion (82–84). NF-κB also enhances chromatin accessibility at FoxP3 and CTLA-4, reinforcing Treg stability (85).

Hypoxia-induced HIF-1α serves as a crucial link between tumor metabolism and immune suppression in CRC (86). HIF-1α upregulates glycolytic enzymes and immunosuppressive genes such as ARG1, CD73, and PD-L1 (87), and directly binds the FoxP3 promoter to promote Treg differentiation. By regulating HDACs and KDM3A, HIF-1α further embeds these suppressive programs through epigenetic mechanisms (88, 89).

**Figure 2:**
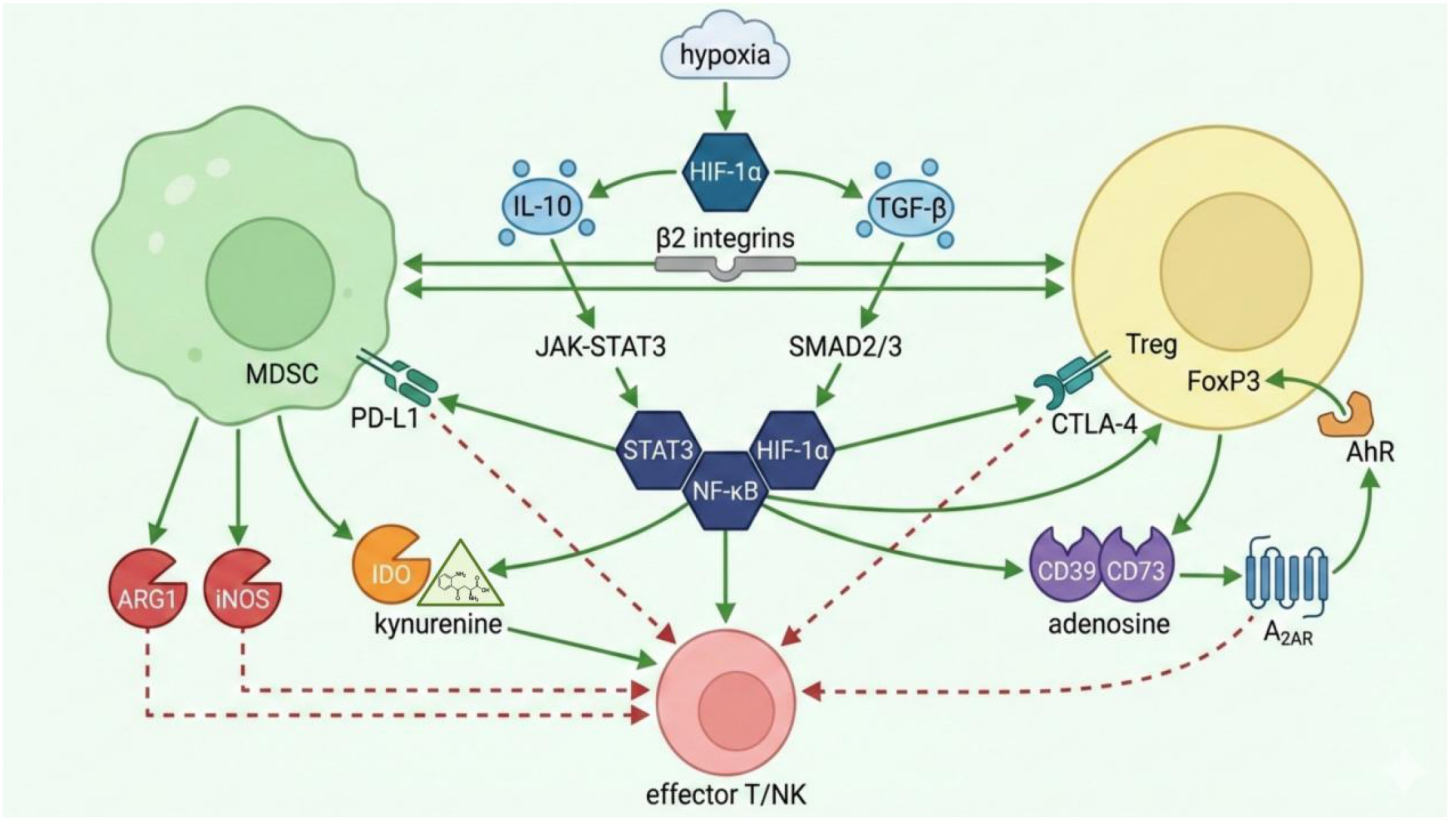
Core molecular mechanisms sustaining the MDSC–Treg immunosuppressive axis in CRC. Created with BioRender.com.

An integrated schematic overview illustrating the core cytokine, metabolic, contact-dependent, and transcriptional mechanisms sustaining the MDSC–Treg immunosuppressive axis in CRC is presented in **Figure 2**.

Schematic illustration depicting the bidirectional and interconnected pathways driving durable immunosuppression. Bidirectional cytokine loops (IL-10 and TGF-β) and contact-dependent interactions (β_2_ integrins, PD-L1/PD-1, CTLA-4) mutually reinforce MDSC and Treg suppressive phenotypes. Metabolic reprogramming includes ARG1/iNOS-mediated arginine depletion, IDO–kynurenine–AhR signaling promoting FoxP3 expression, and CD39/CD73-dependent adenosine generation via the ATP–adenosine–A2AR pathway, all leading to effector T-cell/NK-cell inhibition. These processes converge on central transcriptional hubs STAT3, NF-κB, and HIF-1α (activated by upstream signals including hypoxia, IL-10, TGF-β, and inflammatory mediators), which transcriptionally upregulate immunosuppressive molecules (PD-L1, ARG1, iNOS, CD39/CD73) and stabilize suppressive programs through epigenetic modifications. Solid green arrows indicate activation/induction; dashed red lines indicate inhibition/suppression.

## 4 Tumor microenvironmental factors cooperatively shape the MDSC–Treg immunosuppressive axis

In CRC, the gut-associated TME introduces additional layers of regulation. The microbiota and its metabolites further reinforce the MDSC–Treg axis, making this interaction a central driver of immune escape.

### 4.1 Physicochemical stress and metabolic reprogramming: hypoxia, lactate, and nutrient competition

Hypoxia, a hallmark of the CRC tumor microenvironment, stabilizes HIF-1α, which — as detailed in Sections 3.2 and 3.3 — upregulates key immunosuppressive molecules (PD-L1, ARG1, iNOS, CD73) in MDSCs and promotes FoxP3 expression and peripheral Treg differentiation (90–93). This generates a self-reinforcing immunosuppressive loop that further stabilizes the MDSC–Treg axis under low-oxygen conditions.

In terms of metabolism, the Warburg effect causes high lactate buildup and extracellular acidification. This creates a biochemical environment that strongly supports immunosuppression (94, 95). Lactate helps MDSC expansion and ARG1/iNOS induction, and it also serves as a metabolic source that keeps Treg function stable (64, 94, 96). At the same time, glucose loss caused by high lactate use weakens CD8^+^ T-cell energy metabolism and leads to functional exhaustion (97, 98). This metabolic competition reduces antitumor immunity and gives MDSCs and Tregs a selective advantage, which leads to a stable suppressive microenvironment.

### 4.2 Gut microbiota and microbial metabolites as CRC-specific modulators

CRC is closely linked to gut ecology, so the TME is strongly shaped by microbial signals (99, 100). Microbial components and metabolites change immune signaling and metabolism. They also increase the number and suppressive strength of MDSC and Treg cells (101). The main groups—oncogenic bacteria, beneficial bacteria, and key metabolites—act through direct receptor signals, metabolite-driven changes, and immune-cell recruitment. In this way, they form a “microbiota–metabolism–immunity” loop.

#### 4.2.1 Oncogenic species: activators of the MDSC–Treg axis

Fusobacterium nucleatum (Fn) is one of the most well-known CRC-related bacteria. It contributes to immune evasion, TME changes, and therapy resistance (102, 103). Fn colonization increases intratumoral CD11b^+^Gr-1^+^ MDSC and Foxp3^+^ Treg infiltration (104). Its outer-membrane protein Fap2 engages TIGIT or Gal-GalNAc to suppress NK and T-cell cytotoxicity (105), while FadA activates Wnt/β-catenin signaling in tumor cells, promoting inflammatory and immunosuppressive mediators (106). Fn also stimulates TLR4/NF-κB/MAPK signaling in macrophages, inducing S100A8/A9 and favoring M2 polarization, which further recruits and activates MDSC (107, 108). Collectively, Fn acts as a key microbial driver of the MDSC–Treg axis.

Peptostreptococcus anaerobius is enriched in CRC mucosa and patient stool (109). Its surface protein PCWBR2 binds integrin α2/β1 on tumor epithelium, activating PI3K–Akt–NF-κB signaling and promoting inflammatory and suppressive-cell infiltration (110). Recent evidence shows that P. anaerobius diminishes responses to PD-1 blockade (111) and enhances TLR2/TLR4 cooperation, ROS production, cholesterol synthesis, and epithelial dysplasia in murine models (112).

#### 4.2.2 Beneficial species: potential to counteract the MDSC–Treg axis

Clostridium butyricum enhances anti-PD-1 efficacy in CRC (113) and suppresses tumor development by modulating Wnt signaling and reshaping the microbial community (114). Its surface protein SecD binds GRP78 on CRC cells, inactivating GRP78 and the PI3K–Akt–NF-κB pathway, thereby reducing IL-6 secretion (115). This decrease in IL-6 indirectly limits MDSC expansion and dampens Treg-mediated suppression.

#### 4.2.3 Microbial metabolites: context-dependent modulators

4-HPA, a tyrosine-derived aromatic metabolite elevated in CRC (116), is taken up by tumor cells and activates JAK2/STAT3 signaling, inducing CXCL3 and recruiting CXCR2^+^ PMN-MDSC to the tumor bed (117). Targeting CXCL3 or reducing 4-HPA enhances the efficacy of PD-1 blockade, highlighting its role as a metabolic link between microbiota and MDSC function (117).

Short-chain fatty acids (SCFAs) such as butyrate generally exhibit anti-inflammatory activity by suppressing IL-6/IL-12 and inducing IL-10/IL-16, thereby promoting Treg differentiation (118, 119). However, in tumors, butyrate and propionate may increase Treg frequencies and weaken responses to CTLA-4 blockade, reflecting their context-dependent dual roles (120).

Similarly, inosine, a purine-derived metabolite produced by certain beneficial commensal bacteria such as Bifidobacterium pseudolongum, exerts context-dependent immunomodulatory effects that can enhance antitumor immunity (121). In preclinical models of CRC and other tumors, inosine activates the adenosine A2A receptor on T cells, promoting Th1 cell differentiation and effector function in the presence of inflammatory signals or ICB therapy (121, 122). This metabolite cooperates with checkpoint inhibitors to augment CD8^+^ T-cell responses and improve therapeutic efficacy, potentially by counteracting immunosuppressive barriers in the TME. Clinical evidence further supports the protective role of butyrate-producing bacteria, which are associated with reduced risk of hospitalization for infectious diseases, underscoring the broader health benefits of microbiota-derived metabolites that favor immune activation over suppression (123).

Secondary bile acids, including deoxycholic acid (DCA), are elevated in CRC and suppress CD8^+^ T-cell cytotoxicity by inhibiting PMCA activity and limiting Ca²^+^–NFAT2 signaling (124). Although direct evidence for their regulation of MDSC or Treg is limited, current data suggest that DCA may influence myeloid differentiation and immunosuppressive phenotypes through FXR and TGR5 signaling.

### 4.3 Exosome-mediated intercellular communication expands the immunosuppressive network

Exosome-mediated communication is a central mechanism by which the MDSC–Treg axis propagates and amplifies across the CRC microenvironment (125). Tumor cells, MDSCs, and Tregs all release exosomes carrying regulatory miRNAs, proteins, and soluble mediators, enabling long-range dissemination of immunosuppressive signals within local tissues and the peripheral circulation (126).

In CRC, tumor cells exposed to hypoxia or high lactate release exosomes enriched in miR-21, miR-146a, TGF-β1, and Galectin-9, which drive the differentiation of bone marrow precursors toward MDSCs and enhance their suppressive capacity, while simultaneously promoting Treg expansion or stabilization (127–129). These tumor-derived vesicles create a “TEX–MDSC–Treg” loop that increases the suppressive environment. Several immunoregulatory miRNAs, such as miR-21, miR-146a, and miR-494, also make MDSC and Treg suppressive functions stronger by targeting pathways like PTEN/STAT3 and NF-κB (130, 131).

Microbiota and hypoxia-driven immunosuppression converge on shared effectors (STAT3/NF-κB activation, IL-10/TGF-β, PMN-MDSC/CCR5^+^ Treg recruitment), yet differ in initiation and dominance. Oncogenic bacteria and metabolites (e.g. Fn via TLR4–NF-κB/Fap2–TIGIT, 4-HPA→JAK2/STAT3–CXCL3) trigger early inflammatory recruitment, while hypoxia/HIF-1α provides sustained metabolic amplification (CD73/A2AR, ARG1/iNOS, FoxP3) in established tumors. This suggests early-stage microbiota modulation (probiotics/antibiotics) and advanced-stage hypoxia targeting (HIF inhibitors/anti-angiogenesis) may offer complementary therapeutic windows in CRC.

**Figure 3:**
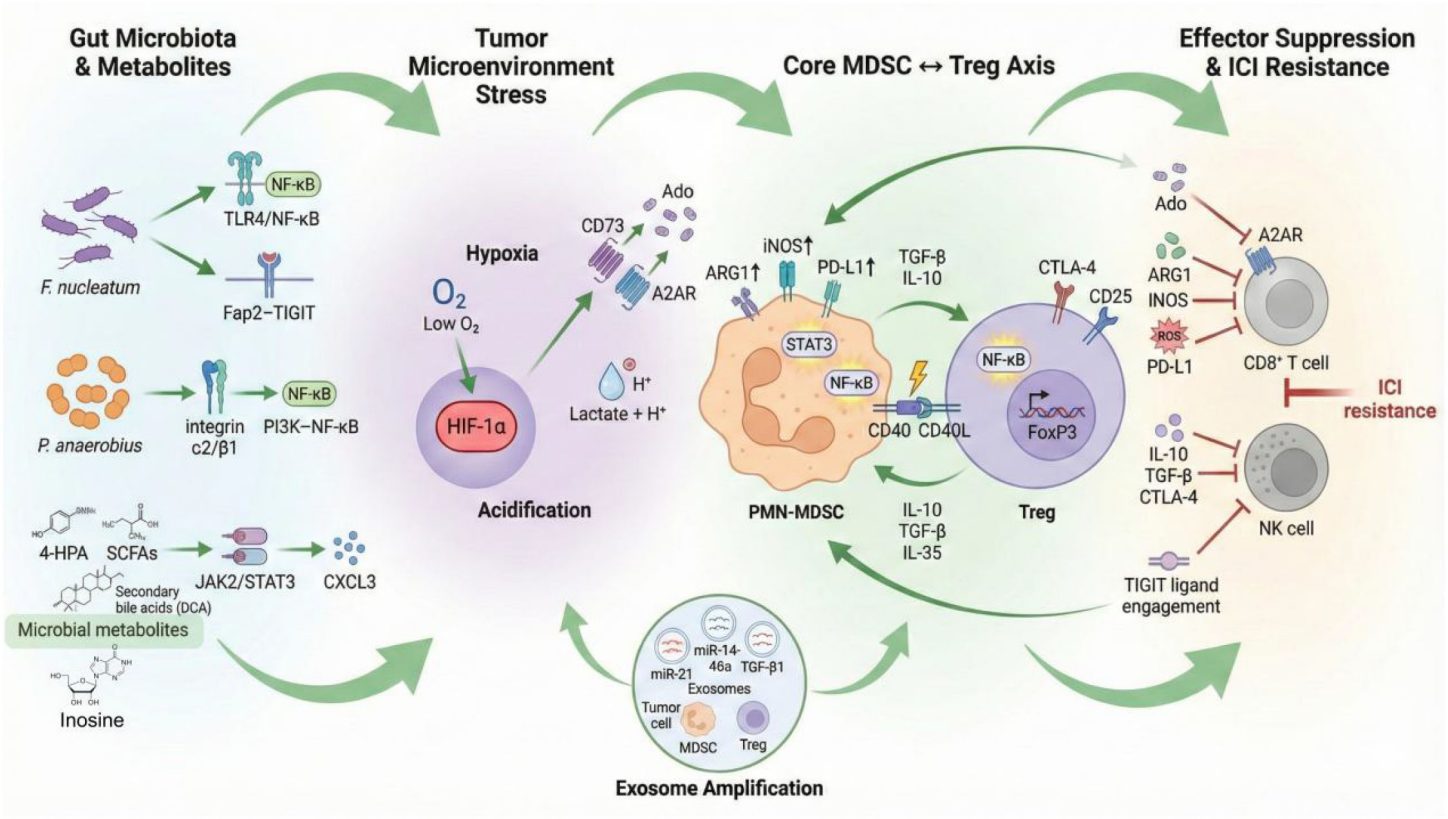
Integrated microbiota–metabolism–hypoxia–MDSC–Treg immunosuppressive cascade in microsatellite-stable/proficient mismatch-repair (MSS/pMMR) CRC. Created with BioRender.com.

An integrated schematic illustrating the microbiota–metabolism–hypoxia–MDSC–Treg immunosuppressive cascade is shown in **Figure 3**.

Key tumor-microenvironmental factors shaping the MDSC–Treg axis are summarized in **Table 1**.

**Table 1:** Tumor microenvironmental factors shaping the MDSC–Treg immunosuppressive axis in CRC.

Category	Key factor	Mechanism in CRC	Effect on MDSC/Treg	Ref.
4.1 Stress & Metabolism	Hypoxia (HIF-1α)	Upregulates PD-L1, ARG1, iNOS, CD73; promotes FoxP3	Reinforces immunosuppressive loop	(90–93)
	Lactate/Acidosis	Fuels MDSC/Treg function; depletes glucose for CD8^+^ T cells	Creates privileged niche for suppression	(94–98)
4.2 Microbiota	Fusobacterium nucleatum	Fap2 binds TIGIT; FadA activates Wnt/β-catenin; TLR4 signaling	↑ MDSC/Treg infiltration; ↓ NK/T-cell killing	(102–108)
	Peptostreptococcus anaerobius	Activates PI3K–Akt–NF-κB via integrin binding	↑ Suppressive cells; ↓ anti-PD-1 response	(109–112)
	Clostridium butyricum	Inactivates GRP78/PI3K pathway, reduces IL-6	Limits MDSC expansion; enhances anti-PD-1	(113–115)
4.2 Metabolites	4-HPA	Activates JAK2/STAT3→CXCL3	Recruits PMN-MDSCs	(116, 117)
	SCFAs (Butyrate)	Promotes Treg differentiation (context-dependent)	May increase Tregs in tumors	(118–120)
	Inosine	Activates A2AR on T cells	Enhances Th1/CD8^+^ T cells with ICB	(121, 122)
4.3 Exosomes	Tumor/MDSC exosomes	Carry miR-21, -146a, TGF-β, Galectin-9	Drives MDSC differentiation; expands Tregs	(125–131)

Oncogenic gut bacteria (Fusobacterium nucleatum via TLR4–NF-κB and Fap2–TIGIT; Peptostreptococcus anaerobius via integrin α2/β1–PI3K–NF-κB) and microbial metabolites (e.g., 4-hydroxyphenylacetic acid [4-HPA] activating JAK2/STAT3–CXCL3) drive the recruitment and expansion of PMN-MDSCs and regulatory T cells (Tregs). Tumor hypoxia and lactate accumulation stabilize HIF-1α, which upregulates CD73/A2AR adenosine signaling and further reinforces the suppressive phenotypes of both MDSCs (ARG1, iNOS, PD-L1) and Tregs (FoxP3, CTLA-4). Bidirectional reinforcement between MDSCs and Tregs is mediated by cytokines (TGF-β, IL-10, IL-35), CD40–CD40L contact, and shared activation of STAT3/NF-κB pathways. Exosomes released from tumor cells, MDSCs, and Tregs carrying miR-21, miR-146a, and TGF-β amplify the axis distally. The net result is profound suppression of effector CD8^+^ T cells and NK cells, leading to an immune-excluded/desert phenotype and resistance to immune checkpoint inhibitors.

## 5 Therapeutic strategies targeting the MDSC–Treg axis and clinical advances

Given that the interaction between MDSCs and Tregs in the TME is a main driver of the “immune desert” or “immune-excluded” states in MSS/pMMR CRC (132, 133), PD-1/PD-L1 blockade alone often has limited effect (134). Recent clinical studies show that treatments that try to break this immunosuppressive axis—by reducing the source of these cells, lowering their suppressive activity, or changing the metabolic environment—can help turn “cold tumors” into “hot tumors” (133, 135). This section reviews therapies that target the MDSC–Treg axis and the related clinical progress.

### 5.1 Inhibition of cell recruitment and angiogenesis: cutting off the axis at its source

Chemokines and angiogenic factors are key signals that guide MDSC and Treg movement into tumors. Blocking these signals can lower the number of immunosuppressive cells from the start. A major example is the phase III STELLAR-303 trial (136), It showed that the multi-target TKI Zanzalintinib plus Atezolizumab (a PD-L1 inhibitor) increased overall survival in patients with refractory MSS CRC (median OS 10.9 vs 9.4 months; HR 0.80, 95% CI 0.69–0.93; P = 0.0045) (136). Zanzalintinib blocks TAM kinases (TYRO3, AXL, MER), MET, and VEGFR at the same time, and this can lower MDSC and Treg levels (137). This treatment offers the first phase III evidence of better immunotherapy outcomes in MSS CRC. It also shows that blood-vessel-related side effects, like high blood pressure, should be watched.

Other approaches use anti-angiogenic agents together with PD-1/PD-L1 inhibitors. In the single-arm phase II CAPability-01 study (n=34), Sintilimab (anti-PD-1) plus Chidamide (HDAC inhibitor) and Bevacizumab (anti-VEGF) reached an ORR of 44% (95% CI 25.5%–63.9%) in previously treated MSS/pMMR metastatic CRC (138). The results look good, but the small sample and lack of a control group mean the findings still need larger studies. These combinations likely function by reducing tumor angiogenesis and associated hypoxia, thereby attenuating HIF-1α-driven immunosuppressive programs (see Sections 3.3 and 4.1), enhancing effector T-cell infiltration, and decreasing MDSC/Treg accumulation (139).

Importantly, angiogenesis-centered strategies represent only one facet of source-directed intervention. Increasing attention has been directed toward therapies that directly modulate myeloid cell differentiation and survival, particularly those targeting the CSF-1/CSF-1 receptor (CSF-1R) axis.

#### 5.1.1 Targeting the CSF-1/CSF-1R axis: reprogramming the myeloid compartment in CRC

The CSF-1/CSF-1R axis is a key regulator of myeloid cell survival, differentiation, and immunosuppressive programming in the CRC TME (140). Tumor cells, stromal fibroblasts, and endothelial cells express elevated CSF-1, which recruits and sustains monocytic MDSCs and M2-like TAMs—both major facilitators of Treg infiltration, angiogenesis, and cytotoxic T-cell suppression (140, 141). CSF-1R activation drives downstream signaling that promotes myeloid survival while blocking maturation into immunostimulatory dendritic cells (142).

Preclinical studies in CRC and other solid tumors show that CSF-1R inhibition (e.g., pexidartinib/PLX3397, BLZ945) reduces MDSC and TAM abundance and function, with concomitant decreases in intratumoral Tregs (143, 144). Importantly, blockade induces functional reprogramming, shifting TAMs from M2-like immunosuppressive to M1-like antigen-presenting phenotypes, thereby disrupting cytokine- and metabolite-mediated feedback loops between myeloid cells and Tregs (145).

In CRC models, CSF-1R inhibitors exhibit limited monotherapy efficacy, consistent with their primary role as immune modulators rather than direct cytotoxics (146). However, combination with PD-1/PD-L1 blockade markedly enhances antitumor activity by alleviating myeloid suppression, reducing IL-10/TGF-β and ARG1/iNOS levels, and boosting CD8^+^ T-cell infiltration and effector function (144).

Early-phase trials of CSF-1R inhibitors in solid tumors, including CRC, demonstrate manageable safety but modest response rates as monotherapy, reinforcing the view that these agents serve best as platforms for TME reprogramming (147, 148). Current efforts therefore focus on rational combinations with immune checkpoint inhibitors, anti-angiogenic agents, or multi-target TKIs to achieve more durable remodeling of the immunosuppressive niche (149).

### 5.2 Novel immune checkpoint combinations: relieving synergistic suppression

Other than PD-1 and CTLA-4, new checkpoints such as TIM-3, TIGIT, and LAG-3 also help maintain the MDSC–Treg axis. These molecules interact with ligands (e.g., Galectin-9, MHC-II) to cooperatively inhibit T cell activation and effector function, stabilizing the immunosuppressive network (150–153). In CRC TME, tumor-infiltrating CD4^+^ T cells upregulate PD-1, CTLA-4, TIM-3, and LAG-3, with high co-expression in the highly suppressive CD4^+^FoxP3^+^Helios^+^ Treg subset. FoxP3^high Helios^+^ Tregs express higher levels of CTLA-4, TIM-3, and LAG-3 compared with FoxP3^low T cells, conferring stronger suppressive potential (154). This heterogeneity highlights the central role of Treg subsets in IC-mediated resistance, suggesting that depleting high IC-expressing Tregs should be prioritized to reverse TME immunosuppression.

Multiple early studies have evaluated the safety and preliminary activity of the combination of anti-TIM-3 monoclonal antibodies (such as Cobolimab/TSR-022, Sabatolimab/MBG453) with PD-1 inhibitors (155–157). AMBER et al. Phase I/Ib studies have shown that this combination regimen has controllable safety and observable anti-tumor activity in various solid tumors, but there is still limited publicly available evidence of benefits for MSS-CRC (155, 156).

In CRC TME, immunophenotyping and histology indicate TIGIT is frequently expressed on tumor-infiltrating T cells, including FoxP3^+^ Tregs, often co-expressed with other inhibitory checkpoints, suggesting a role in maintaining intratumoral immunosuppression (158, 159). Clinically, anti-TIGIT antibody Tiragolumab combined with anti-PD-L1 Atezolizumab has shown early activity in other cancers such as NSCLC (160). Analyses of CRC tumor samples revealed that this combination increased Th1/Treg and Tc1/Treg ratios, indicating an association between high cytotoxic T cells, low immunosuppressive cells, and combination therapy (161). However, efficacy in MSS/pMMR CRC remains unconfirmed in large, reproducible cohorts.

LAG-3, an inhibitory checkpoint expressed on activated CD4^+^/CD8^+^ T cells, Tregs, and NK cells (162, 163), suppresses antigen-specific T cell activation and proliferation in CRC TME while enhancing Treg stability and immunosuppressive function (164). Clinically, Relatlimab (anti-LAG-3) combined with Nivolumab (PD-1 inhibitor) has shown durable benefit in MSI-H/dMMR metastatic CRC (165). In the CheckMate 142 trial (NCT02060188), this combination achieved an ORR of 50% (95% CI 36%–65%) in previously treated MSI-H/dMMR patients, with manageable safety (mainly diarrhea and fatigue) (165).

### 5.3 Stratified therapeutic approaches based on microsatellite status

MSI-H/dMMR CRC remains sensitive to PD-1 inhibitors but resistance occurs in some patients. Single-cell transcriptomic analyses reveal enrichment of MDSCs, particularly PMN-MDSCs, in resistant tumors, suggesting compensatory MDSC upregulation contributes to PD-1 resistance (166). Clinically, low-dose Ipilimumab (1 mg/kg) combined with Nivolumab yielded an ORR of ~55% in dMMR/MSI-H metastatic CRC (167). In first-line cohorts, the combination achieved an ORR of ~69% in phase II studies (168).

For MSS/pMMR CRC, removing Tregs alone cannot reverse the strong immunosuppression. So, “dual” or “multi-step” strategies are needed. These include TKI combinations or new checkpoint co-blockade. They aim to block MDSC recruitment and also weaken Treg function. In this way, they can better overcome immune resistance.

### 5.4 Disrupting the MDSC–Treg axis to enhance vaccine-based and other immunotherapeutic modalities

Beyond immune checkpoint blockade, disrupting the MDSC–Treg axis holds promise for improving vaccine-based immunotherapies in MSS/pMMR CRC, where “cold” tumors often limit antigen-specific T-cell priming and effector function (169). Dendritic cell (DC) vaccines aim to overcome this by loading autologous DCs with tumor antigens to stimulate antitumor immunity. The GEMCAD 1602 Phase I/II trial evaluated an autologous DC vaccine combined with avelumab (anti-PD-L1) in pretreated MSS metastatic CRC patients, demonstrating favorable safety and modest clinical efficacy, including immune activation in a subset of cases (170). Although primary endpoints were not fully met, these results highlight the potential of DC vaccines in pMMR disease when combined with strategies to alleviate immunosuppression.

Preclinical evidence indicates that MDSCs and Tregs directly impair vaccine efficacy by suppressing DC maturation, antigen presentation, and effector T-cell responses (171, 172). Depletion or reprogramming of these cells can synergize with vaccines: for instance, CSF-1R inhibitors reduce MDSC/TAM accumulation, enhancing CD8^+^ T-cell infiltration and vaccine-induced immunity in multiple tumor models (146, 173). Low-dose cyclophosphamide selectively depletes Tregs, amplifying responses to peptide or whole-cell vaccines (e.g., GVAX) (174, 175). Emerging modalities, such as neoantigen vaccines or viral vector-based platforms, may similarly benefit from axis disruption to convert immunosuppressive niches into supportive environments for durable T-cell memory (169).

Integrating MDSC/Treg-targeted agents (e.g., CXCR2 antagonists, all-trans retinoic acid, or STAT3 inhibitors) with vaccines represents a rational approach to sensitize MSS/pMMR CRC, potentially extending beyond ICI combinations (176, 177).

A summary of the main therapeutic strategies that target the MDSC–Treg axis, along with related clinical evidence, is shown in **Table 2**.

**Table 2:** Therapeutic strategies targeting the MDSC–Treg axis in CRC and corresponding clinical advances.

Therapeutic strategy	Key mechanisms & targets	Example agents/Combinations	Clinical trial & key findings	Ref.
5.1 Inhibition of Recruitment & Angiogenesis	Blocks chemokine/angiogenic signals to reduce MDSC/Treg infiltration and tumor hypoxia.	Zanzalintinib (multi-TKI) + Atezolizumab	STELLAR-303 (Ph III): Improved OS in refractory MSS-CRC (mOS 10.9 vs 9.4 mos; HR 0.80).	(136, 137)
		Sintilimab (anti-PD-1) + Chidamide + Bevacizumab	CAPability-01 (Ph II, n=34): ORR 44% in pretreated MSS/pMMR mCRC.	(138, 139)
5.1.1 Targeting CSF-1/CSF-1R	Reprograms myeloid cells, reduces monocytic MDSC/M2-TAM survival, decreases Tregs.	CSF-1R inhibitors (e.g., Pexidartinib, BLZ945) ± PD-1/PD-L1 inhibitors	Preclinical: Reduces MDSC/TAM/Tregs, enhances CD8+ T-cell activity. Clinical early-phase: Best as combination platform for TME reprogramming.	(140–149)
5.2 Novel Immune Checkpoint Combos	Blocks synergistic suppression from checkpoints highly expressed on Tregs.	Anti-TIM-3 (e.g., Cobolimab) + PD-1 inhibitor	Early-phase studies: Manageable safety, anti-tumor activity in solid tumors; limited specific data for MSS-CRC.	(150–157)
		Anti-TIGIT (Tiragolumab) + Atezolizumab	Early activity in NSCLC; in CRC samples, increased Th1/Tc1 to Treg ratios. Efficacy in MSS-CRC unconfirmed.	(158–161)
		Anti-LAG-3 (Relatlimab) + Nivolumab	CheckMate 142: ORR 50% in pretreated MSI-H/dMMR mCRC. Role in MSS/pMMR not established.	(162–165)
5.3 Stratified by MS Status	MSI-H/dMMR	Nivolumab + Low-dose Ipilimumab	ORR ~55-69% in metastatic dMMR/MSI-H CRC.	(166–168)
	MSS/pMMR	Requires “dual/multi-step” strategies (e.g., TKI combos, novel co-blockade) to target both MDSC recruitment and Treg function.	Strategies aim to overcome strong, multi-faceted immunosuppression.	(Context)
5.4 Enhancing Vaccine/Other Modalities	Disrupts axis to improve antigen presentation and effector T-cell function in “cold” tumors.	DC vaccine + Avelumab (anti-PD-L1)	GEMCAD 1602 (Ph I/II): Favorable safety, modest efficacy, immune activation in some pretreated MSS mCRC.	(169, 170)
		Vaccines + MDSC/Treg-targeting agents (e.g., low-dose cyclophosphamide, CSF-1Ri, STAT3i)	Preclinical: Depleting/reprogramming MDSCs/Tregs synergizes with vaccines to enhance T-cell immunity and memory.	(171–177)

## 6 Conclusion and future perspectives

Clinical translation is accelerating. The phase III STELLAR-303 trial demonstrated a clear overall survival benefit with zanzalintinib plus PD-L1 inhibition in refractory MSS CRC, supported by consistent activity in anti-angiogenic + ICI combinations. These findings position VEGF pathway inhibition combined with PD-(L)1 blockade and multi-target TKIs affecting MDSC/Treg recruitment as the most evidence-based and near-term strategies. In parallel, novel checkpoint inhibitors targeting TIM-3, TIGIT, and LAG-3 show early promise, particularly given their high expression on highly suppressive Treg subsets, highlighting the potential for more precise targeting. By contrast, metabolic interventions (IDO, CD73/A2AR, ARG1 inhibitors), CSF-1R blockade, microbial metabolite modulation, and exosome-targeted approaches remain mechanistically compelling but largely preclinical or early-phase, awaiting CRC-specific validation.

Challenges persist: most novel checkpoint and immunomodulatory data derive from phase I/II trials, and functional, spatial, metabolic, and response heterogeneity within MDSC–Treg subsets remains incompletely understood. Patient selection also requires improved immune profiling and predictive tools.

Future advances will likely emerge from:

1. better phenotypic analyses using single-cell, multi-omics, and spatial immune methods;
2. more precise immunotherapy plans based on differences within Treg and MDSC subsets;
3. improved combinations of new checkpoints, anti-angiogenic drugs, TKIs, and epigenetic modulators;
4. useful biomarkers that can predict benefit or resistance to combination therapy;
5. microbiome-based immune strategies, including probiotics, postbiotics, and microbial metabolites that can reshape the gut–tumor immune axis.

Overall, with deepening mechanistic insights and ongoing high-quality clinical research, therapeutic strategies targeting the MDSC–Treg axis hold promise to become a new pillar of immunotherapy in MSS/pMMR CRC. In the future, multi-mechanism, multi-targeted, and individualized combination regimens are likely to be key for achieving deeper responses and durable survival, offering new solutions to the longstanding challenge of immune resistance in MSS CRC.
